# Parental Social Isolation during Adolescence Alters Gut Microbiome in Rat Male Offspring

**DOI:** 10.3390/biom14020172

**Published:** 2024-01-31

**Authors:** Carlotta Siddi, Sofia Cosentino, Elena Tamburini, Luca Concas, Maria Barbara Pisano, Riccardo Ardu, Maura Deplano, Paolo Follesa, Elisabetta Maciocco, Patrizia Porcu, Mariangela Serra, Maria Giuseppina Pisu

**Affiliations:** 1 Department of Life and Environmental Sciences, Section of Neuroscience and Anthropology, University of Cagliari, Monserrato, 09042 Cagliari, Italy; carlottasiddi@yahoo.it (C.S.); concasluca93@gmail.com (L.C.); follesa@unica.it (P.F.); 2 Department of Medical Sciences and Public Health, University of Cagliari, Monserrato, 09042 Cagliari, Italy; scosenti@unica.it (S.C.); barbara.pisano@unica.it (M.B.P.); mdeplano@unica.it (M.D.); 3 Department of Biomedical Sciences, University of Cagliari, Monserrato, 09042 Cagliari, Italy; etamburini@unica.it (E.T.); riccardo.ardu@unica.it (R.A.); 4 Neuroscience Institute, National Research Council of Italy (CNR), Monserrato, 09042 Cagliari, Italy; elisabetta.maciocco@in.cnr.it (E.M.); patrizia.porcu@in.cnr.it (P.P.); mariagiuseppina.pisu@in.cnr.it (M.G.P.)

**Keywords:** social isolation, preconceptional stress, male rats offspring, gut microbiome, cytokines, corticosterone

## Abstract

Previous work from our laboratory demonstrated that parental stress, induced by social isolation starting at puberty, leads to behavioral, endocrine, and biochemical changes in the male, but not female, offspring (ISO-O) of Sprague-Dawley rats. Here, we report alterations in the gut microbiota composition of ISO-O vs. grouped-housed offspring (GH-O), although all animals received the same diet and were housed in the same conditions. Analysis of bacterial communities by next-generation sequencing (NGS) of 16S rRNA gene revealed alterations at family and order levels within the main phyla of Bacteroides, Proteobacteria, and Firmicutes, including an almost total deficit in *Limosilactobacillus reuteri* (formerly *Lactobacillus reuteri*) and a significant increase in *Ligilactobacillus murinus* (formerly *Lactobacillus murinus*). In addition, we found an increase in the relative abundance of Rhodospirillales and Clostridiales in the families of Lachnospiraceae and Ruminococcaceae, and Bacteroidales in the family of Prevotellaceae. Furthermore, we examined plasma levels of the proinflammatory cytokines interleukin-1-beta and tumor necrosis factor-alpha, which did not differ between the two groups, while corticosterone concentrations were significantly increased in ISO-O rats. Our findings suggest that adverse environmental conditions experienced by parents may have an impact on the likelihood of disease development in the subsequent generations.

## 1. Introduction

It is generally accepted that early life experiences have complex and profound effects on brain development and maturation, including the induction of persistent plasticity of the neuroendocrine stress system [1,2]; furthermore, the pathophysiological effects are not limited to the affected individual, given that transgenerational influence of the stressful experience can be extended across multiple generations [3,4]. In contrast to the plethora of studies on the effect of stress-induced adverse events occurring in the perinatal period, the influence of chronic stress experienced by parents during adolescence on subsequent neurodevelopment in their offspring needs to be further explored.

Our laboratory has described relationships between long-lasting stressful experiences, such as post-weaning social isolation, and the occurrence of a plethora of biochemical and behavioral alterations in male offspring [5,6]. Post-weaning social isolation has been shown to be a relevant animal model for studying the mechanisms underlying psychopathological states induced by stressful experiences [7]. Our early studies have shown that post-weaning social isolation induces, in male adult animals, biochemical and behavioral effects indicative of a state of anxiety and depression [8,9,10,11]. Additionally, social isolation in female rats decreased the quality of maternal care that these dams provide to their young [12]. Poor maternal care has been widely considered a critical impact on development. Accordingly, male offspring of socially isolated rats (ISO-O), despite being raised in a group, showed social deficits, behavioral perseveration, and increased seizure sensitivity, lower levels of the “social” neuropeptide oxytocin, higher levels of brain-derived neurotrophic factor (BDNF) and the neurosteroid allopregnanolone in both brain and plasma, increased basal hypothalamic-pituitary-adrenal (HPA) axis activity, along with a blunted reactivity to acute stress [5,6] (see also Table 1 for a summary). Female offspring were tested for behavioral perseveration, seizure sensitivity, oxytocin, and BDNF plasma levels and showed a normal phenotype [6].

Accumulating evidence indicates a link between the microbiota-gut-brain axis and the stress responses, pointing out that the underlying mechanisms are complex and bidirectional [13]. Moreover, post-weaning social isolation in rodents modifies gut microbiota, thereby disrupting normal neural, endocrine, metabolic, and immunological bidirectional communication between the gut microbiome and the brain [14,15].

Gut microbiota is crucial for brain development and function, being essential for maintaining homeostasis and plasticity [16] and also affecting social behavior, cognition, and mood; indeed, both clinical and preclinical studies have demonstrated significant changes in the composition of gut microbiota in psychiatric disorders associated with neurodevelopment and neurodegeneration [17].

Based on these premises, we hypothesized that ISO-O male rats might have an altered gut microbiota despite being raised in a group without any stress exposure during their life. Therefore, by using 16S ribosomal RNA gene sequencing (16S rRNA sequencing), we investigated putative changes in microbial richness and composition in ISO-O male rats at weaning (PND 21) and in adulthood (PND 60), given that the microbiota displays plasticity throughout the life course [18]. In addition, given the evidence that stress hormones may boost the immune response through the induction of proinflammatory cytokines such as interleukin-1beta (IL-1β) and tumor necrosis factor-alpha (TNF-α) [19], we investigated putative alterations in peripheral levels of these two proinflammatory mediators in ISO-O males and their mothers. 

## 2. Materials and Methods

### 2.1. Animals 

Experiments were performed in the male offspring of Sprague-Dawley rats or in their mothers, both obtained from our colony generated from breeders purchased from Charles River (Calco, Italy). All animals were maintained under an artificial 12/12 h light/dark cycle at a constant temperature of 23 ± 2 °C and 65% humidity. Food and water were freely available at all times. Adequate measures were taken to minimize the pain or discomfort of animals whose care and handling throughout the experimental procedures were in accordance with the European Parliament and the Council Directive of 22 September 2010 (2010/63/EU) and were approved by the Italian Ministry of Health (Authorization no. 1122/2016-PR), according to the Italian Legislative Decree no. 26 of 4 March 2014. We made every effort to minimize pain and suffering and to reduce the number of animals used.

### 2.2. Generation of Offspring from Socially Isolated Parents 

Male and female Sprague-Dawley rats at 21 days of age, immediately after weaning, were housed for 30 days either in groups of five per cage (59 × 38 × 20 cm; group-housed, GH) or individually in smaller cages (42 × 26 × 15 m; socially isolated, ISO). For the breeding procedure, 51-day-old males and females were paired for 5 days; ISO females were bred with ISO males, and GH females were bred with GH males in order to match the same protocol previously described by Pisu et al. [5]. The day in which sperm was detected in the vaginal smear was designated as gestational day 0 (G0). Upon gestational status identification, female rats were either singly housed or group-housed (depending on which experimental group they belonged to, ISO or GH) until gestational day 20, when every rat was singly housed for parturition and subsequent nursing. Male and female offspring of GH and ISO parents (GH-O and ISO-O, respectively) were weaned 21 days after birth and then housed in groups of five per cage. Rats housed in the same cage belonged to different litters, such that each litter provided 2/3 males in order to avoid any litter effect. Experiments were performed at PND 21 and PND 60.

### 2.3. Sample Collection, Storage, and DNA Extraction

To collect fecal boluses, ISO-O and GH-O rats on PND 21 and PND 60 were numbered on the tail and were moved to a clean housing cage. They were constantly monitored from 9:00 a.m. to 12:00 p.m. by two different experimenters, who immediately collected fresh stool samples into sterile 2 mL tubes, which, upon collection, were stored at −80 °C until their processing for DNA extraction (*n* = 5 per experimental group). For DNA extraction, about 2 mg of feces were placed into a sterile 1.5 mL microcentrifuge tube containing 1 mL of sodium citrate (2%, pH 7.5) and centrifuged at 10,000× *g* for 30 min at 4 °C. Then, 1000 μL lysis buffer (200 mM Tris-HCl, pH 8.0, 250 mM NaCl, 25 mM EDTA, SDS 0.5% *w*/*v*) was added to the pellet, and the tube was vigorously vortexed for 1 min. A total of 200 μL of the sample were then transferred to another tube, and 50 μL of SDS (10% *w*/*v*) and 18 μL of proteinase K solution (5 mg/mL, Sigma-Aldrich Italia, Milan, Italy) were added. The sample was vigorously vortexed and incubated for 60 min at 65 °C before the addition of 100 μL 2.5 M potassium acetate (Sigma-Aldrich Italia, Milan, Italy). The tubes were slowly inverted, placed at −20 °C for 2 h, and then centrifuged at 11,000× *g* for 30 min at 4 °C. A total of 300 μL of the supernatant was transferred to a new tube, and an equal volume of 2-propanol (Sigma-Aldrich Italia, Milan, Italy) was added to precipitate nucleic acids. The tubes were left to stay at room temperature for 5 min and then were centrifuged at 11,000× *g* for 15 min at 4 °C. The resulting DNA pellet was washed with 500 µL 70% ethanol, and the tubes were centrifuged again at 11,000× *g* for 15 min. Afterward, the DNA pellet was air-dried and resuspended in 120 μL TE buffer (10 mM Tris-HCl, pH 8.0, 1 mM EDTA). DNA concentration and purity were checked by optical density at 260 nm, and ratio O.D. 260 nm/280 nm was determined using the microplate spectrophotometer SPECTROstar^®^ Nano equipped with LVIS Plate (BMG LABTECH, Ortenberg, Germany). The DNA concentration of the fecal boluses was in the range of 3.25 ng/µL–11.74 ng/µL, as requested by the external company that carried out the library construction and sequencing.

### 2.4. Analysis of Bacterial Communities by Next-Generation Sequencing (NGS) of 16S rRNA Gene

DNA samples were submitted to BMR Genomics (Padua, Italy) for the sequencing of the V3–V4 region of the bacterial 16S rRNA gene on an Illumina Miseq platform (Illumina, San Diego, CA, USA) using 2 × 300 bp paired-end reads. For data processing, raw sequences were demultiplexed by the sequencing facility. 

Sequences were imported into Quantitative Insights into Microbial Ecology (QIIME 2) version 2018-11. Reads were trimmed to remove primer sequences, suitable-quality reads were dereplicated and denoised, discharging ambiguous and poor-quality sequences, and the paired reads were merged using the DADA2 plugin [20]. Chimeras and singletons were identified and removed. DADA2 was used to produce amplicon sequence variants (ASVs), and a filtered ASV-abundance table was obtained. For each ASV, taxonomy assignment to the lowest taxonomic level (i.e., from genus to phylum) was performed against the Silva database release 132.

The indices of diversity (richness as the number of observed ASV, Shannon index with an e log base) and evenness (Pielou’s index) were used to assess the alpha diversity. Read count data were normalized by cumulative sum scaling (CSS) transformation using the metagenomeSeq package, version 1.43.0. An ordination analysis of the bacterial communities based on the Bray-Curtis dissimilarity index between samples was performed by non-metric multidimensional scaling (nMDS) in the phyloseq package (version 1.46.0). Permutational multivariate analysis of variance (PERMANOVA) was then used to evaluate the null hypothesis that there were no significant differences between pairs of groups in the experimental design (age: PND 21 versus PND 60; parents’ housing condition: ISO-O versus GH-O). PERMANOVA was performed using the adonis function in the vegan package, version 2.6.4, on the Bray-Curtis dissimilarity matrix with 9999 permutations.

### 2.5. Interleukins and Corticosterone Assays 

Animals (*n* = 10 per experimental group) were sacrificed by decapitation. Blood was collected into K3-EDTA tubes, centrifuged at 1000× *g* for 10 min at 4 °C, and plasma was collected into tubes and frozen at −80 °C until use. The enzyme-linked immunosorbent assay (ELISA) was used to quantify plasma levels of IL-1β and TNF-α (#ELR-IL1β-1 and #ELR-TNF-α-1; RayBiotech, Peachtree Corners, GA, USA), as well as corticosterone (#RE52211; Tecan, Mannendorf, Switzerland). ELISA assays were performed according to the manufacturer’s instruction using a 96-well plate pre-coated with a polyclonal antibody against the respective analyte of interest.

### 2.6. Statistical Analysis 

The ASVs contribution to the observed Bray-Curtis similarity with respect to the two factors (age and parents’ housing condition) was investigated by means of a two-way crossed SIMPER analysis as implemented in Primer 7 software version 7.0.13. The two-way analysis of variance (ANOVA) followed by Tukey’s post hoc test (GraphPad Prism 8 Software, San Diego, CA, USA) was used to compare the means of the indices of diversity and evenness as well as the relative abundance of each single taxon across each of two factors in the experimental design (age and parents’ housing condition) and their interaction. Interleukins and corticosterone levels are presented as means ± SD, and results were analyzed by two-tailed unpaired Student’s *t*-test. For all data analyzed, a value of *p* < 0.05 was considered statistically significant.

## 3. Results

### 3.1. Structure of Bacterial Communities

**Figure 1:**
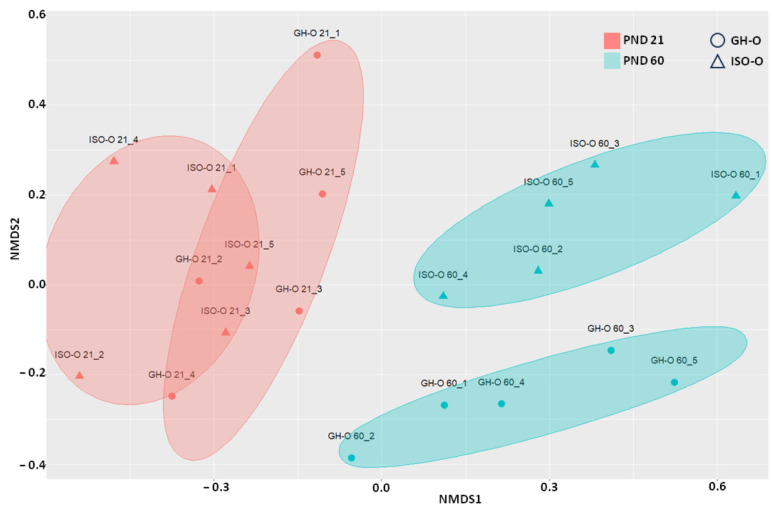
Non-metric multidimensional scaling (nMDS) of bacterial communities in fecal boluses of ISO-O (triangles) and GH-O rats (circles).

Alpha diversity indices (Table 2) did not significantly differ between groups (two-way ANOVA *p* > 0.05). The nMDS plots constructed using the Bray-Curtis dissimilarity index (Beta diversity) are shown in Figure 1. Multivariate analyses demonstrated that “age” was the main factor affecting the structure of bacterial communities (PERMANOVA *p* = 0.001). A significant difference in the fecal microbiota was also revealed between ISO-O and GH-O rats (PERMANOVA *p* = 0.007). Moreover, the interaction of the two experimental factors was found to be significant (PERMANOVA *p* = 0.014).

### 3.2. Shift in Composition of Bacterial Communities in ISO-O Rats

**Figure 2:**
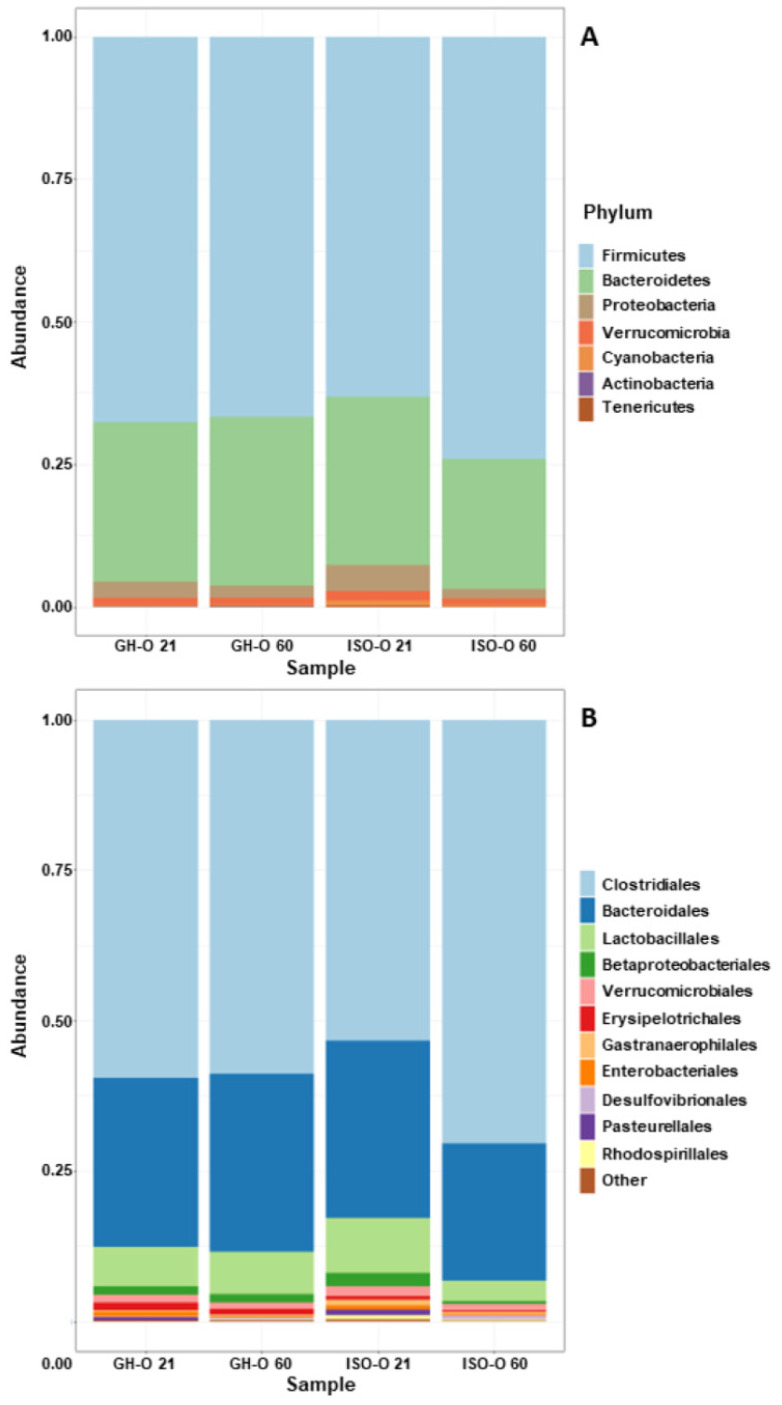
Composition of the fecal microbiota in ISO-O and GH-O rats. The relative abundances of the top 10 phyla Panel (**A**) and orders Panel (**B**) in bacterial communities are shown.

**Figure 3:**
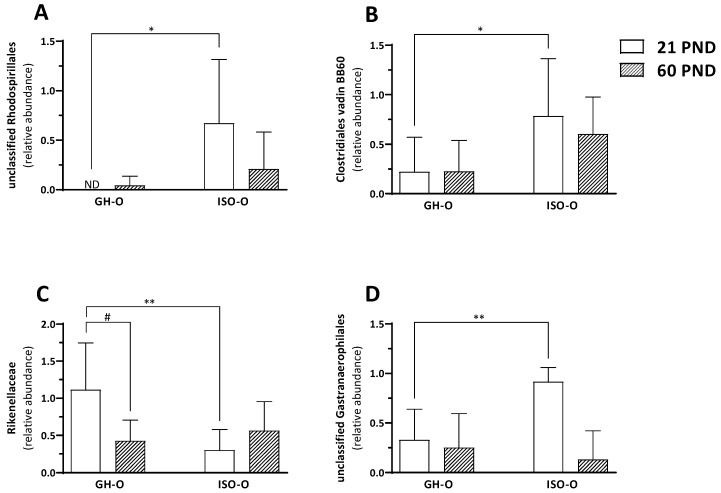
Relative abundances of an unclassified family of Rhodospirillales Panel (**A**), Clostridiales vadin BB60 group Panel (**B**), Rikenellaceae Panel (**C**), and an unclassified family of Gastranaerophilales Panel (**D**) in the fecal microbiota of ISO-O and GH-O rats. Data are the mean ± SD of 5 animals per group. * *p* < 0.05, ** *p* < 0.01 vs. the respective age-matched GH-O group; ^#^ *p* < 0.05 vs. PND 21 GH-O rats (two-way ANOVA followed by Tukey’s post hoc test).

**Figure 4:**
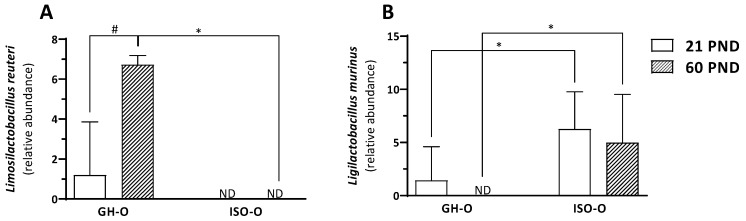
Relative abundance of two ASVs attributed to the genera *Limosilactobacillus* and *Ligilactobacillus* (formerly *Lactobacillus*) in the fecal microbiota of ISO-O and GH-O rats: *Limosilactobacillus reuteri* Panel (**A**) (ASV10, Table 3) and *LigiLactobacillus murinus* Panel (**B**) (ASV13, Table 3). Data are the mean ± SD of 5 animals per group. * *p* < 0.01 vs. the respective age-matched GH-O group; ^#^ *p* < 0.01 vs. PND 21 GH-O rats (two-way ANOVA followed by Tukey’s post hoc test).

In investigating the shift in the composition of the fecal microbiota in the ISO-O rats, we compared the relative abundance of a single taxon from the phylum to the genus level across each of the two factors of the experimental design (age and parents’ housing condition) and the interactions between them. At the phylum level, no significant differences were found between GH-O and ISO-O animals (Figure 2A). Despite a reduction in the Bacteroidetes/Firmicutes ratio in PND 60 ISO-O rats compared to age-matched GH-O controls, such an effect was not statistically significant (33.1% vs. 47.3%, respectively, *p* = 0.167); further, no differences were found between PND 21 ISO-O and GH-O (47.3% vs. 41.8%, respectively, *p* = 0.562). At order level (Figure 2B), ANOVA revealed a significant effect of parents’ housing condition only on Erysipelotrichales (F(1,16) = 5.264, *p* = 0.036), no significant effect of age (F(1,16) = 1.628, *p* = 0.220), and no interaction between factors (F(1,16) = 0.004, *p* = 0.952); post hoc comparison did not reveal any significant difference between groups. At the family level, ANOVA revealed a significant difference in parents’ housing conditions for the relative abundance of the unclassified family of Rhodospirillales (F(1,16) = 6.248, *p* = 0.024, Figure 3A). The relative abundance of the taxon was also significantly higher in the ISO-O group at PND 21 with respect to age-matched GH-O controls (*p* = 0.012), while no significant difference based on age (F(1,16) = 1.581, *p* = 0.226) and no interaction between factors (F(1,16) = 2.274, *p* = 0.151) were found. A significant effect of parents’ housing condition (F(1,16) = 6.354, *p* = 0.023), no significant effect of age (F(1,16) = 0.232, *p* = 0.637), and no interaction between factors (F(1,16) = 0.260, *p* = 0.617) were highlighted for the Clostridiales vadin BB60. More specifically, its relative abundance was significantly higher in PND 21 ISO-O rats compared to the respective GH-O group (*p* = 0.048) (Figure 3B). For the Rikenellaceae family (Figure 3C), ANOVA revealed no significant effect of parents’ housing condition (F(1,16) = 3.254, *p* = 0.090), no significant effect of age (F(1,16) = 1.255, *p* = 0.279), but a significant interaction between factors (F(1,16) = 6.391, *p* = 0.022). Post hoc analysis showed a significant reduction in ISO-O rats with respect to age-matched GH-O controls at PND 21 (*p* = 0.007). The relative abundance of an unclassified family in Gastranaerophilales of the phylum Cyanobacteria was also altered in ISO-O rats. ANOVA revealed a significant effect of age (F(1,16) = 11.698, *p* = 0.003), no significant effect of parents’ housing condition (F(1,16) = 3.391, *p* = 0.084), and a significant interaction between factors (F(1,16) = 7.868, *p* = 0.013) (Figure 3D); the unclassified Gastranaerophilales was increased in ISO-O rats at PND 21 with respect to age-matched GH-O controls (*p* = 0.004). At the genus level, the relative abundances of 10 taxa were found to be significantly different between the two parents’ housing conditions, and the results of their two-way ANOVA statistics are shown in Table 3. The genus *Pygmaiobacter*, the uncultured group GCA-900066225 (in the family of Ruminococcaceae), and an uncultured group in Rhodospirillales were significantly increased in the ISO-O group compared to GH-O controls across all ages considered. For these three taxa, no significant effect of age and no significant interaction between factors were found. Higher relative abundances for the genera *Ruminiclostridium* 5 and *Acetatifactor* were also found in ISO-O rats compared to GH-O controls across all considered ages. Specifically, ANOVA revealed significant effects for the two factors (i.e., parents’ housing condition and age) and a significant interaction between factors. In contrast, the relative abundances of the genera *Turicibacter*, *Erysipelatoclostridium* and *Alistipes*, and an uncultured *Muribaculaceae* were significantly lower in the ISO-O group as compared to the GH-O control group across all considered ages. For these four taxa, a significant effect of parents’ housing condition, no significant effect of age, and no interaction between factors were found. The relative abundance of *Ruminococcaceae* UCG-005 was lower in the ISO-O group compared to GH-O controls across all ages. In particular, ANOVA revealed a significant effect of parents’ housing conditions and a significant effect of age. At the species level, the relative abundances of two ASVs attributed to the genera *Limosilactobacillus* and *Ligilactobacillus* were found to be significantly different between ISO-O and GH-O rats (Figure 4A,B and Table 4). Specifically, the ASV10, related to *L. reuteri*, was not detected at all in fecal boluses of ISO-O rats either at PND 21 or at PND 60 (*p* = 0.0001), and its relative abundance increased with age in control GH-O rats (*p* = 0.00008). Two-way ANOVA revealed a significant effect of parents’ housing condition (F(1,16) = 42.639, *p* < 0.0001), a significant effect of age (F(1,16) = 20.801, *p* = 0.0003), and a significant interaction between factors (F(1,16) = 20.801, *p* = 0.0003). For ASV13, phylogenetically related to the *L. murinus,* ANOVA revealed a significant effect of parents’ housing condition (F(1,16) = 11.156, *p* = 0.004), no significant effect of age (F(1,16) = 0.844, *p* = 0.372), and no interaction between factors (F(1,16) = 0.002, *p* = 0.961). ISO-O rats showed a higher relative abundance at PND 21 and PND 60 compared to age-matched GH-O controls (*p* = 0.033 and *p* = 0.029, respectively). Further, the relative abundance of *L. murinus* decreased with age in control rats and was not detected in GH-O at PND 60.

The ASVs contribute the most to differences in fecal microbiota composition between groups as identified by the two-way SIMPER analysis, and the results of their two-way ANOVA statistics are shown in Table 4. In addition to the two mentioned above, three ASVs attributed to the taxa Prevotellaceae, Muribaculaceae, and *Ruminococcus* 1 also differed significantly between the ISO-O and GH-O groups. In particular, ANOVA revealed a significant effect of parents’ housing condition (F(1,16) = 11.100, *p* = 0.004), no significant effect of age (F(1,16) = 1.548, *p* = 0.231), and no interaction (F(1,16) = 0.250, *p* = 0.624) for ASV01 (attributed to Prevotellaceae group NK3B31), which was increased in the ISO-O group compared to GH-O controls across all ages considered (*p* = 0.004). A significant effect of parents’ housing condition (F(1,16) = 20.270, *p* = 0.0004), a significant effect of age (F(1,16) = 7.889, *p* = 0.013), and no significant interaction between factors (F(1,16) = 0.496, *p* = 0.491) were found for ASV08 (attributed to Muribaculaceae), and its relative abundance decreased in ISO-O with respect to GH-O rats across all ages (*p* = 0.0004). Finally, ASV15 (attributed to *Ruminococcus* 1) was also altered in ISO-O rats; ANOVA revealed a significant effect of parents’ housing condition (F(1,16) = 7.419, *p* = 0.015), no significant effect of age (F(1,16) = 2.120, *p* = 0.165) and no significant interaction between factors (F(1,16) = 1.304, *p* = 0.270), with ASV15 being more abundant in the ISO-O group compared to GH-O controls across all ages considered (*p* = 0.01).

### 3.3. Inflammatory Pattern and Corticosterone Levels in Plasma of Socially Isolated Dams and Male ISO-O at PND 21 

The plasma concentration of IL-1β and TNF-α in socially isolated (ISO) and group-housed (GH) female rat dams was evaluated on postpartum day 21. As shown in Table 5, ISO dams did not show significant differences in plasma levels of IL-1β and TNF-α compared to GH rats ((t(18) = 0.936, *p* = 0.369) and (t(18) = 0.163, *p* = 0.873), respectively). Furthermore, plasma levels of IL-1β and TNF-α were also evaluated in their male offspring at PND 60, and no differences were observed between GH-O and ISO-O rats ((t(18) = 0.369, *p* = 0.717) and (t(18) = 0.980, *p* = 0.343), respectively).

Basal corticosterone levels were also measured in ISO dams, and male ISO-O was measured at PND 60. ISO dams showed a significant decrease in corticosterone levels with respect to GH control dams (−45%; t(18) = 4.927, *p* = 0.001); on the contrary, plasma corticosterone was increased in male ISO-O, compared to GH-O rats (+24%; t(18) = 2.546, *p* = 0.02) (Table 5).

## 4. Discussion

We report that parental stress, induced by social isolation starting at puberty, leads to changes in the gut microbiome composition in their male F1 offspring. To our knowledge, this is the first report on changes in microbiome composition that occur in the offspring of parents that were subjected to chronic stress during adolescence. A previous study has described the influence of paternal diet and maternal care on gut microbiome composition in the offspring that was also exposed to the predator odor acute stress. These offspring animals showed altered weight, anxiety-like behavior, and a shift in Firmicutes to Bacteroidetes ratio [21]. Our ISO-O rats were reared under standard conditions and were never subjected to stress during the experimental procedures; however, they received reduced maternal care [12]. Although previous studies have described no influence of maternal care on offspring’s microbiome, this possibility cannot be ruled out; indeed, additional evidence from carefully designed and well-powered studies is needed to clarify whether alterations in the quality of maternal care may affect gut microbes in the adult offspring. In rodents, a decrease in the quality and frequency of maternal care has been shown to affect behavior, emotions, and stress response in the offspring [22,23,24]. However, male ISO-O shows an attenuated corticosterone release following acute foot-shock stress but no alterations in emotional reactivity [5] or in spatial learning and memory performances [6]. Therefore, the quality of maternal care is not the only determinant for the phenotype observed in male ISO-O; future studies will be necessary to establish the maternal or paternal contribution to the offspring programming in our model. One of the most noticeable results found in our study is the total absence of *L. reuteri* in the gut of ISO-O rats at PND 21 and PND 60. *L. reuteri* naturally colonized a wide range of vertebrates, humans and rodents included [25], and studies using specific supplementation strategies have related this probiotic bacterium to immunomodulation, neuromodulation, and neurodevelopmental disorders [26]. Reduced levels of *L. reuteri* were reported in autism spectrum disorder (ASD) models such as high-fat diet (HFD) offspring, BTBR T+ Itpr3tf/J (BTBR), and Shank3B^−/−^ mice [27,28]. *L. reuteri* increased the central and peripheral expression and secretion of oxytocin, the hormone implicated in the neurobiological alterations that occur in ASD. Accordingly, the same studies demonstrated that treatment with *L. reuteri* reversed the social behavior and social behavior-related plasticity deficits in these ASD mouse models and in Cntnap2^−/−^ mice in an oxytocin-dependent manner [27,28]. In agreement, our ISO-O rats with undetectable levels of *L. reuteri*, also showed reduced plasma oxytocin levels, together with decreased agonistic behavior and social transmission of flavor preference [6]. In contrast, the abundance of *L. murinus* was significantly increased in ISO-O rats at both ages tested. Importantly, administration of *L. murinus* HU-1 was sufficient to rescue behavioral deficits and brain region-specific microglial activation observed in maternal microbiome dysbiosis-reared murine offspring [29]. Hence, the high proportion of *L. murinus* in male ISO-O might act as a compensatory mechanism downstream of the social behavior deficit previously reported in ISO-O rats [6]. Moreover, these rats showed a significant increase in the relative abundance of a single unclassified family in Rhodospirillales that represents a single unclassified family belonging to the *α*-Proteobacteria class. This result is consistent with that found in rat male offspring whose mothers were treated with valproic acid during pregnancy [30]. 

Modulation of behavior may occur through both direct and indirect signaling by the gut microbiota. Short-chain fatty acids (SCFAs) are considered one key candidate for the role of direct mediators [31]. Numerous preclinical and clinical studies suggest that SCFAs perform multiple central and peripheral functions; however, the heterogeneity in terms of study design and study population has not led to an unambiguous conclusion on the direction of modulation. The most abundant SCFAs are acetate, propionate, and butyrate [32]; propionate and butyrate are produced in the colon by Clostridiales [33] and Erysipelotrichales [34]. In the feces of ISO-O rats, we found a significant increase in the abundance of the Clostridiales vadin BB60 group at PND 21 and, although not statistically significant, a decrease in the abundance of Erysipelotrichaceae (−55% at PND 21 and −76% at PND 60, compared to the respective GH-O controls), as well as undetectable levels of the genera *Erysipelatoclostridium* e *Turicibacter* in Erysipelotrichaceae. We can hypothesize that these variations could translate into alterations in the balance levels of these SCAFs that, on the basis of preclinical and clinical studies, could be related to the biochemical and behavioral alterations seen in ISO-O rats (see Table 1 for a summary). For instance, in men, an SCFA mixture attenuated the cortisol response to acute psychosocial stress [31]; likewise, an attenuation in corticosterone release following acute foot-shock stress was also observed in ISO-O male animals [5]. Clostridiaceae are a relatively large family of bacteria, and their diversity makes it difficult to identify specific markers that could link the increase in Clostridiaceae family members to different disease states. However, some species of *Clostridium* produce propionate whose intracerebroventricular injection into the rat brain causes biological and chemical alterations including seizures [33]. It is tempting to hypothesize a correlation between the increase in several taxa belonging to the order Clostridiales observed in the present study and the previously reported increased susceptibility to isoniazid-induced seizures in ISO-O rats [6]. The SCFA butyrate, also produced by Erysipelotrichaceae [34], among the wide array of biological properties, influences brain function by regulating gene expression through inhibitors of histone deacetylases [35]. In addition, it has recently been shown that pre-reproductive stress increased DNA methylation in the amygdala of F1 male offspring [36]. Moreover, sodium butyrate attenuates social behavior deficits [37], and the decreased levels of bacterially produced butyrate are related to epigenetic changes in neurons from Parkinson’s disease patients and to the severity of their depressive symptoms [38]. Individuals with major depression show hyperactivity of the HPA axis with elevated plasma adrenocorticotropic hormone (ACTH) and cortisol concentrations under basal conditions and a blunted ACTH response in the corticotropin-releasing factor (CRF) stimulation test [39,40], the latter of which has been attributed to reduced CRFR1 expression in the anterior pituitary, secondary to chronic hypersecretion of CRF from the hypothalamus. The significant increase in basal levels of plasma corticosterone and the reduction in the expression of CRFR1 in the pituitary of ISO-O rats compared to GH-O rats is thus consistent with the notion that the basal activity of the HPA axis is increased in these animals [5]. Although a direct measurement of depressive-like behavior in ISO-O rats has not been performed, these animals show behavioral features of this pathology in humans, such as an impairment in cognitive flexibility [6,41]. In agreement, adult male rat offspring whose mothers were exposed to chronic unpredictable stress prior to conception showed lower sucrose consumption and an index of anhedonia [42]. On the other hand, some taxa belonging to the families of Prevotellaceae (NK3B31 group) and Ruminococcaceae (*Ruminococcus* 1, CGA-900066225, *Pygmaiobacter*, *Ruminiclostridium* 5) were found to be more abundant in ISO-O rats than in GH-O controls, despite opposite evidence of a lower abundance of these families in the gut of patients with major depression [43,44]. However, the abundance of Prevotellaceae was significantly increased in anhedonia-susceptible mice, compared with a sham group, following spared nerve injury [45]; likewise, Ruminococcaceae UCG-005, whose abundance is significantly reduced in ISO-O male rats, is indicated as the most important genus in predicting depressive symptoms in human [44]. Finally, it has been shown that increasing the abundance of Muribaculaceae, also decreased in ISO-O male rats, can achieve therapeutic antidepressant effects [46]. Nonetheless, further studies are needed to clarify the contribution of these genera to major depression in humans and in animal models.

SCFAs may also exert anti-inflammatory and immunomodulatory effects [47]. In our model, we did not find differences in plasma levels of the proinflammatory cytokines IL-1β and TNF-α between pre-adolescent ISO-O and GH-O male rats and their respective ISO and GH mothers. In contrast, an increased expression of TGF-β in the dorsal hippocampus and of IL-1β and IL-6 in the ventral hippocampus of socially isolated female rats has recently been reported [15]. However, we cannot rule out the possibility of differential regulation in brain vs. plasma levels for these proinflammatory cytokines. Further, to avoid any stress to our pregnant rats, we did not measure levels of proinflammatory cytokines during pregnancy; thus, we cannot exclude that maternal inflammation might also account for the biochemical and behavioral alterations in ISO-O male rats; future more detailed studies are required to explore this hypothesis. 

In conclusion, the current study demonstrated that juvenile social isolation-induced psychosocial stress altered the gut microbiome composition in the subsequent generation, reared under normal conditions, and never exposed to stress. Characterizing stress phenotypes at multiple levels, as well as their severity and timing, is crucial for articulating measurable targets of preventive interventions.

## Figures and Tables

**Table 1:** Biochemical and behavioral changes in ISO-O vs. GH-O male rats at PND 60.

Parameter	Effect
Allopregnanolone brain and plasma levels	Increased [5]
BDNF brain and plasma levels	Increased [6]
Oxytocin plasma levels	Decreased [6]
Basal HPA axis activity	Increased [5]
Acute stress responsiveness	Decreased [5]
Social behavior	Decreased [6]
Behavioral flexibility	Decreased [6]
Seizures sensitivity	Increased [6]
Anxiety-like behavior	No change [5]
Spatial learning and memory	No change [6]
Novelty-induced behavior	No change [6]
Aggressive behavior	No change [6]
Locomotor activity	No change [5]

Data are from Pisu et al., 2013 [5] and Pisu et al., 2019 [6].

**Table 2:** Alpha diversity of bacterial communities in fecal boluses of GH-O and ISO-O rats at PND 21 and PND 60 estimated by the indices of diversity and evenness.

Groups	S	H’	J’
GH-O PND 21			
1	99	4.548031	0.9897524
2	114	4.680060	0.9881470
3	143	4.903395	0.9880212
4	110	4.653042	0.9899079
5	152	4.966281	0.9885349
GH-O PND 60			
1	117	4.704156	0.9878169
2	74	4.253327	0.9882116
3	173	5.092157	0.9881367
4	129	4.798470	0.9873776
5	138	4.872007	0.9887875
ISO-O PND 21			
1	110	4.642027	0.9875644
2	73	4.245236	0.9894596
3	101	4.559047	0.9878501
4	87	4.420237	0.9897734
5	132	4.831808	0.9895565
ISO-O PND 60			
1	131	4.822543	0.9891996
2	147	4.924567	0.9868017
3	120	4.735935	0.9892309
4	122	4.747505	0.9882356
5	138	4.874141	0.9892206

The indices of diversity indices (S: richness as the number of observed ASVs; H’: Shannon index with an e log base) and evenness (J’: Pielou’s) were used to evaluate alpha diversity and statistically tested by two-way ANOVA.

**Table 3:** Taxa at the genus level in fecal boluses significantly different between GH-O and ISO-O rats in two-way ANOVA analysis.

Taxonomy			Examines Age Groups (across All Type Groups)		Examines Type Groups (across All Age Groups)		Two-Way ANOVA					
Order	Family	Genus	PND 21	PND 60	GH-O	ISO-O	Type		Age		Interaction	
							F(1,16)	*p*	F(1,16)	*p*	F(1,16)	*p*
Bacteroidales	Muribaculaceae		1.16 ± 0.59	0.97 ± 0.62	1.35 ± 0.42	0.77 ± 0.63	6.37	**0.02**	0.67	0.42	2.70	0.12
	Rikenellaceae	*Alistipes*	0.34 ± 0.39	0.40 ± 0.24	0.51 ± 0.32	0.23 ± 0.25	5.33	**0.03**	0.26	0.62	3.55	0.08
Clostridiales	Lachnospiraceae	*Acetatifactor*	0.00 ± 0.00	0.31 ± 0.42	0.00 ± 0.00	0.31 ± 0.42	11.50	**0.004**	11.50	**0.004**	11.50	**0.004**
	Ruminococcaceae	GCA-900066225	0.25 ± 0.37	0.24 ± 0.27	0.05 ± 0.11	0.44 ± 0.33	10.51	**0.005**	0.014	0.91	0.018	0.89
	Ruminococcaceae	*Pygmaiobacter*	0.13 ± 0.27	0.10 ± 0.21	0.00 ± 0.00	0.23 ± 0.30	5.13	**0.04**	0.10	0.75	0.10	0.75
	Ruminococcaceae	*Ruminiclostridium* 5	0.46 ± 0.51	1.39 ± 0.99	0.47 ± 0.48	1.37 ± 1.02	14.20	**0.002**	15.23	**0.001**	9.12	**0.008**
	Ruminococcaceae	UCG-005	1.80 ± 0.79	0.75 ± 0.59	1.69 ± 0.68	0.85 ± 0.87	10.48	**0.005**	16.75	**0.0009**	0.0029	0.96
Erysipelotrichales	Erysipelotrichaceae	*Erysipelatoclostridium*	0.26 ± 0.42	0.04 ± 0.14	0.30 ± 0.42	0.00 ± 0.00	6.62	**0.02**	3.38	0.08	3.38	0.08
	Erysipelotrichaceae	*Turicibacter*	0.24 ± 0.43	0.04 ± 0.14	0.28 ± 0.42	0.00 ± 0.00	5.40	**0.03**	2.63	0.12	2.63	0.12
Rhodospirillales			0.31 ± 0.51	0.08 ± 0.15	0.02 ± 0.07	0.37 ± 0.49	6.15	**0.02**	2.51	0.13	3.55	0.08

Data are the mean ± SD of 5 animals per group. Bold: two-way ANOVA, *p* < 0.05.

**Table 4:** Two-way SIMPER analysis of ASVs in fecal boluses of GH-O and ISO-O rats at PND 21 and PND 60.

ASV	Taxonomy			Two-Way SIMPER								Two-Way ANOVA					
				Examines Age Groups (across All Type Groups)				Examines Type Groups (across All Age Groups)				Type		Age		Interaction	
No.	Family	Genus	Species	PND 21 (ab ± SD)	%	PND 60 (ab ± SD)	%	GH-O (ab ± SD)	%	ISO-O (ab ± SD)	%	F(1,16)	*p*	F(1,16)	*p*	F(1,16)	*p*
ASV05	Bacteroidaceae	*Bacteroides*		5.1 ± 3.5	2.1	3.0 ± 3.9	0.8	5.1 ± 3.6	2.2	3.0 ± 3.9	0.9	1.555	0.23	1.499	0.24	0.437	0.52
ASV07	Bacteroidaceae	*Bacteroides*		5.5 ± 4.7	2.0	8.0 ± 3.0	4.7	8.2 ± 3.0	4.8	5.2 ± 4.6	1.8	2.928	0.11	2.178	0.16	0.016	0.90
ASV11	Bacteroidaceae	*Bacteroides*		4.1 ± 3.6	1.5	2.9 ± 3.8	0.8	4.9 ± 3.5	2.1	2.1 ± 3.4	0.3	3.009	0.10	0.567	0.46	0.051	0.82
ASV03	Muribaculaceae			5.2 ± 2.9	2.8	3.6 ± 3.8	1.0	3.4 ± 3.7	0.9	5.3 ± 3.0	2.9	1.613	0.22	1.123	0.31	0.491	0.49
ASV04	Muribaculaceae			4.7 ± 3.3	2.2	2.3 ± 3.7	0.2	2.8 ± 3.6	0.7	4.2 ± 3.7	1.8	0.820	0.38	2.381	0.14	0.020	0.89
ASV08	Muribaculaceae			2.5 ± 3.2	1.2	5.2 ± 2.9	2.9	6.0 ± 2.2	3.5	1.7 ± 2.8	0.5	20.270	**0.0004**	7.889	**0.01**	0.496	0.49
ASV09	Muribaculaceae			2.5 ± 3.2	0.4	5.7 ± 3.2	3.0	5.1 ± 3.6	2.6	3.1 ± 3.3	0.7	2.206	0.16	5.670	**0.03**	1.468	0.24
ASV14	Muribaculaceae			4.1 ± 2.8	1.8	4.2 ± 3.7	1.5	3.2 ± 3.4	0.8	5.1 ± 2.8	2.5	1.775	0.20	0.006	0.94	0.128	0.73
ASV01	Prevotellaceae	NK3B31		7.4 ± 3.9	4.4	5.5 ± 4.4	2.7	3.9 ± 4.6	1.0	9.0 ± 1.1	6.2	11.100	**0.004**	1.548	0.23	0.250	0.62
ASV10	Lactobacillaceae	*Limosilactobacillus*	*L. reuteri*	0.6 ± 1.9	0.0	3.4 ± 3.6	2.3	4.0 ± 3.4	2.2	0.0 ± 0.0	0.0	42.640	**0.0001**	20.800	**0.0003**	20.800	**0.0003**
ASV13	Lactobacillaceae	*Ligilactobacillus*	*L. murinus*	3.8 ± 4.1	1.7	2.5 ± 4.0	0.8	0.7 ± 2.2	0.0	5.6 ± 3.9	2.5	11.160	**0.004**	0.844	0.37	0.002	0.96
ASV02	Lachnospiraceae	*Blautia*		5.2 ± 3.7	2.8	0.5 ± 1.6	0.0	2.1 ± 3.5	0.2	3.6 ± 3.9	2.7	1.877	0.19	17.710	**0.0007**	5.078	**0.04**
ASV06	Lachnospiraceae			5.4 ± 4.7	2.0	2.7 ± 3.6	0.4	4.6 ± 4.3	2.0	3.4 ± 4.4	0.5	0.453	0.51	2.123	0.16	1.700	0.21
ASV15	Ruminococcaceae	*Ruminococcus* 1		4.2 ± 4.5	2.0	2.0 ± 3.4	0.3	1.0 ± 2.3	0.0	5.2 ± 4.5	2.3	7.419	**0.01**	2.120	0.16	1.304	0.27
ASV12	Akkermansiaceae	*Akkermansia*		5.1 ± 4.5	1.6	5.7 ± 4.1	0.2	5.2 ± 4.6	1.8	5.6 ± 4.0	2.2	0.032	0.86	0.078	0.78	0.062	0.81

Two-way SIMPER analysis of ASVs that were found to have >2% contribution toward the similarity between groups in at least one group. Mean abundance in percentage ± standard deviation (ab ± SD) and percentage contribution to similarity (%) of each taxon (*n*= 5 animals per group). Bold: two-way ANOVA, *p* < 0.05.

**Table 5:** Inflammatory pattern and corticosterone levels in plasma from socially isolated dams and their male ISO-O.

	IL-1β	TNF-α	Corticosterone
	(pg/mL)		(ng/mL)
**Dams**			
GH	629.4 ± 141.8	690.0 ± 235.2	130.0 ± 20.8
ISO	731.0 ± 243.7	709.7 ± 191.5	71.5 ± 21.4 **
**Male offspring PND 60**			
GH-O	539.8 ± 87.1	519.3 ± 106.9	96.2 ± 16.7
ISO-O	609.5 ± 181.2	538.4 ± 107.3	126.2 ± 22.5 *

Socially isolated (ISO) and group-housed (GH) female rats, as well as their respective male offspring (ISO-O and GH-O), were sacrificed for measurement of interleukins and corticosterone plasma concentrations. Data are expressed as pg/mL or ng/mL and represent the mean ± SD of values from 10 rats per group. All data were analyzed by a two-tailed unpaired Student’s *t*-test. * *p* < 0.05, ** *p* < 0.01 vs. the respective controls.

## Data Availability

Data will be available upon request.
